# International ethics harmonization and the global alliance for genomics and health

**DOI:** 10.1186/gm530

**Published:** 2014-02-27

**Authors:** Bartha M Knoppers

**Affiliations:** 1 Centre of Genomics and Policy, McGill University, 740 Dr. Penfield Avenue, Suite 5200, Montreal H3A 0G1, Canada

## 

On 28 January 2013, funders and researchers met to discuss how to enable rapid progress for biomedicine by lowering global barriers to responsible and secure data sharing [1]. Since then, over 130 leading organizations in health care, research and disease advocacy, operating in 40 countries, have signed a letter of intent to work towards the creation of a Global Alliance for Genomics and Health (GA4GH) [2]. There are three goals: to enable open standards for interoperability of technology platforms for managing and sharing genomic and clinical data, to provide guidelines and harmonized procedures for privacy and ethics internationally, and to engage stakeholders to encourage responsible sharing of data and of methods.

After the mapping and sequencing of the human genome, today’s challenge is the integration of genomics data with clinical data. However, interpretation of individual sequences for genomic medicine requires an evidence base of secure data sharing within a framework of core principles. The GA4GH core principles are as follows: respect - protecting secure data sharing and privacy preferences of participants; transparency - ensuring open governance and operations; accountability - promoting best practices in technology, ethics and outreach; inclusivity - partnering and building trust among stakeholders; collaboration - sharing information to advance human health; innovation - developing an ecosystem that accelerates progress; agility - acting swiftly to benefit those with disease.

The vision of the GA4GH then is to promote and facilitate the exchange of extensive data on genomic sequences, including clinical annotations, to accelerate progress in genomic medicine, such as for cancer outcomes and targeted therapy, inherited pediatric diseases, other common non-communicable diseases, infectious diseases, and drug responses. In this short paper, we will focus on the international ethics harmonization challenges that the GA4GH faces.

## Initial goals

For 2014, the short-term organizational goals of the GA4GH are to establish an open and transparent structure with an executive and secretariat. Four Working Groups will produce standards and documents that will address the following areas: genomic data interoperability; security and privacy of data; ethics and regulatory issues; and clinical data. Relationships with cloud computing operators that provide distributed information platforms will allow the launch of transformative demonstration projects, first, to create the technical standards for data sharing and, second, to foster testing of processes for the global harmonization of ethics frameworks.

With respect to the first demonstration project, except for disease-based efforts such as, for example, the International Cancer Genome Consortium (ICGC, http://www.icgc.org/) and the International Rare Diseases Research Consortium (http://www.irdirc.org/), data are still siloed and international standards for informatics are missing or not up to scale. Although security and privacy technologies are increasingly sophisticated, more work needs to be done to create a system that recognizes data producers and managers. Internationally recognized systems for the proper identification of the provenance of bioresources and genomic and clinical data, as well as scientific attribution for accompanying analyses, are emerging [3,4]. Cloud computing operators that agree to be platform development partners need to establish both common standards and tools, including the necessary software, as well as voluntary models of self-regulation and certification. The GA4GH will chiefly mobilize and catalyze these efforts to enable operators and working groups to develop and implement interoperable application program interfaces and technologies.

However, for the second demonstration project, the socio-ethical and legal regulatory challenges of such a global initiative may prove to be formidable. Indeed, although agreement on data standards and quality assurance can (hopefully) be reached with leading information technology centers, companies and data systems managers, consensus on even a standardized set of acceptable processes and procedures for ethical, legal and social implications (ELSI) using the core principles is not certain. Ethics harmonization (as opposed to standardization) may be achievable, however. To accomplish this, the GA4GH will have to maintain both a clear, shared vision and interactive, dynamic communication structures within a global membership that ranges from public and academic, private and patient organizations to policymakers [5]. Harmonizing ELSI tools across these members and countries will not be simple.

## International ethics harmonization

Narrow practices for consent and for privacy protection thwart international exchange. Although international genomics projects such as the ICGC and international consortia such as the Public Population Project in Genomics and Society have worked towards harmonizing ethics approaches, be it for broad consent forms or data and sample access agreements [6], challenges in international uptake remain. Specifically, data are still too often analyzed in isolated disciplinary or institutional silos and with incompatible methods. Regulatory systems still are neither designed nor sufficiently updated to foster widespread cross-study collaboration and trans-border open sharing of data. As such, the continued absence of harmonized ethics norms that can facilitate internationally collaborative global science and responsible research may be the major challenge as the GA4GH attempts to enable the translational transformation of the fruits of public investment in genomics and biomedical research.

Ethics harmonization needs to recognize legal and cultural diversity but remain principled and practical. Thus, the core principles of the GA4GH need to be translated into processes and procedures that facilitate the mutual recognition of ethics review in different countries, avoiding conflicting and inconsistent approaches while retaining the benefits of expert ethics review. We recently proposed a ‘safe harbor’ framework for international ethics review governance [7]. Such a safe harbor model has been used for the recognition of the equivalency of privacy protections following the adoption of the European Privacy Directive in 1995. If adopted for ethics review of data-driven international research projects, it could streamline the current fragmented, inconsistent and inefficient ‘system’. Considering that GA4GH seeks to integrate genomic data with clinical data for research and eventual translation into treatment, such mutual recognition of the adequacy (albeit different) approaches of countries to ethics review is but a first step. The next would be to actually put in place a federated system for ethics review. A cornerstone for such a process would be an international data sharing ‘Code of Conduct’ that could be a beginning for this harmonization process [8]. Cloud computing contributors, producers, operators and users of genomic and clinical data could pledge to adhere to such a Code of Conduct. To ensure maximum effectiveness, the Code could be founded on international human rights policies that are already globally ratified and that currently serve to guide and delimit national laws. A human rights approach would not only bolster already accepted ethics principles [9] but also, more importantly, provide a legal framework for enactment and accountability.

## Next steps

A Code of Conduct could serve to realize what until now have been largely dormant human rights. Indeed, in 1948, article 27 of the *Universal Declaration of Human Rights* proclaimed that ‘Everyone has the right to share in scientific advancements and its benefits … and the right to the protection of the moral and material interests resulting from any scientific … production of which he is the author’ [10]. Herein lies the ideal, to create an inclusive entity that responsibly creates frameworks and tools for genomic and clinical data sharing, fosters the translation of the benefits of research for health, and yet recognizes the contribution of the scientists that make this possible. In short, the GA4GH should engage, encourage and enable, while maximizing ethical harmonization to earn global endorsement and trust.

## Abbreviations

ELSI: Ethical, legal and social implications; GA4GH: Global alliance for genomics and health; ICGC: International cancer genome consortium.

## Competing interests

The author declares that she has no competing interests.
